# EGFR Soluble Isoforms and Their Transcripts Are Expressed in Meningiomas

**DOI:** 10.1371/journal.pone.0037204

**Published:** 2012-05-18

**Authors:** Angélique Guillaudeau, Karine Durand, Barbara Bessette, Alain Chaunavel, Isabelle Pommepuy, Fabrice Projetti, Sandrine Robert, François Caire, Hélène Rabinovitch-Chable, François Labrousse

**Affiliations:** 1 Department of Pathology, Dupuytren University Hospital, Limoges, France; 2 Department of Cellular Homeostasis and Pathology, Dupuytren University Hospital, Limoges, France; 3 Department of Neurosurgery, Dupuytren University Hospital, Limoges, France; 4 Department of Biochemistry and Molecular Genetics, Dupuytren University Hospital, Limoges, France; Instituto de Investigación Sanitaria Incliva, Spain

## Abstract

The EGFR (epidermal growth factor receptor) is involved in the oncogenesis of many tumors. In addition to the full-length EGFR (isoform a), normal and tumor cells produce soluble EGFR isoforms (sEGFR) that lack the intracellular domain. sEGFR isoforms b, c and d are encoded by EGFR variants 2 (v2), 3 (v3) and 4 (v4) mRNA resulting from gene alternative splicing. Accordingly, the results of EGFR protein expression analysis depend on the domain targeted by the antibodies. In meningiomas, EGFR expression investigations mainly focused on EGFR isoform a. sEGFR and EGFRvIII mutant, that encodes a constitutively active truncated receptor, have not been studied. In a 69 meningiomas series, protein expression was analyzed by immunohistochemistry using extracellular domain targeted antibody (ECD-Ab) and intracellular domain targeted antibody (ICD-Ab). EGFRv1 to v4 and EGFRvIII mRNAs were quantified by RT-PCR and EGFR amplification revealed by MLPA. Results were analyzed with respect to clinical data, tumor resection (Simpson grade), histological type, tumor grade, and patient outcome.Immunochemical staining was stronger with ECD-Ab than with ICD-Ab. Meningiomas expressed EGFRv1 to -v4 mRNAs but not EGFRvIII mutant. Intermediate or high ECD-Ab staining and high EGFRv1 to v4 mRNA levels were associated to a better progression free survival (PFS). PFS was also improved in women, when tumor resection was evaluated as Simpson 1 or 2, in grade I *vs.* grade II and III meningiomas and when Ki67 labeling index was lower than 10%.Our results suggest that, EGFR protein isoforms without ICD and their corresponding mRNA variants are expressed in meningiomas in addition to the whole isoform a. EGFRvIII was not expressed. High expression levels seem to be related to a better prognosis. These results indicate that the oncogenetic mechanisms involving the *EGFR* pathway in meningiomas could be different from other tumor types.

## Introduction

Meningiomas are the second most common primary intracranial tumor [1]. According to the World Health Organization (WHO) classification, they consist of grade I (meningothelial, psammomatous, fibroblastic, angiomatous and transitional); grade II (atypical, chordoid and clear cells), which have a high rate of recurrence; and grade III tumors (anaplastic, papillary, rhabdoid), which are highly malignant. Meningiomas infiltrating adjacent brain tissue are considered to be grade II [2].

Epidermal growth factor receptor gene (EGFR/ErbB1) is a member of the ErbB receptor tyrosine kinase family. EGFR overexpression has been reported in a majority of human tumors [3], [4], [5], [6]. Recent therapeutic agents that target EGFR such as monoclonal antibodies and small-molecule tyrosine kinase inhibitors constitute an important progress in various cancer treatments [7], [8], [9], [10] **.**

EGFR is composed of three main domains: an extracellular domain (ECD), a transmembrane domain (TMD), and an intracellular domain (ICD). In addition to the full-lenght transmembrane forms, soluble EGFR (sEGFR) isoforms, that comprised solely the ECD portions of the receptor, have been detected in normal and malignant cells, in tissues, and in biological fluids [11], [12]. These sEGFR proteins can be either generated by alternative mRNA splicing events or via proteolytic cleavage of the receptor [13], [14]. EGFR gene alternative splicing leads to four transcripts: EGFR variants 1, 2, 3 and 4 (v1, v2, v3 and v4, respectively) mRNA that encode 170-kDa whole receptor and 60-kDa [15], 80-kDa [16], [17] and 110-kDa [18] sEGFR isoforms, respectively. Another 110-kDa soluble EGFR isoforms known as PI-sEGFR are produced by proteolytic cleavage triggered in part by metalloproteases [11], [12], [19], [20]. In addition, an aberrant translocation event was found in A431 vulvar carcinoma cell line resulting in the expression of a 115-kDa sEGFR [21]. Circulating sEGFR level have been used as prognosis and theragnosis predictive markers in the serum of patients with cervical [22], colorectal [23], ovarian and breast [24], [25], [26], [27]. The predictive value of sEGFR was also studied directly in tumor tissues from cervical or lung cancer [28], [29].

Since alternative splicing can produce different isoforms, it is critical to know which epitope recognize the antibodies when studying EGFR protein expression. Indeed, others and we reported strong difference in immunohistochemical labeling according to the EGFR domain, ECD or ICD, targeted by primary antibodies [5], [28], [30].

In meningiomas, the role of EGFR signaling pathway in tumor genesis and the usefulness of EGFR investigation in regard to prognosis and/or theragnosis assessment remain unclear and discrepancies exist. Some studies reported higher EGFR protein levels in grade I and grade II meningiomas compared to grade III meningiomas [31], [32]. Smith et al. reported shorter survival times for patients having atypical meningiomas with low EGFR protein levels [33]. Depending on studies, the percentage of meningiomas that overexpress EGFR varied from 40 to 100%, [31], [34], [35], [36], [37], [38], [39]. In addition, the nature of the cells (endothelial or tumor cells), expressing EGFR protein has also been discussed [34], [38], [40], [41]. The lack of consensus in meningiomas regarding EGFR can be attributed to primary antibodies used in immunohistochemistry (IHC) [30], [42] or to primer locations when RT-PCR approaches were used. sEGFR have a potential role in activating or inhibiting the EGFR pathway and their expression pattern can be of major interest for potential therapeutic applications in meningioma [43], [44].

In addition to EGFR overexpression, EGFR gene amplification is another common genetic alteration found in glioma, non small cells lung cancers or colorectal tumors. However, in meningiomas no such alteration was described [40]. EGFR amplification is often associated with the expression of a constitutively active EGFRvIII mutant, in which a portion of ECD is missing. EGFRvIII has been detected in glioblastomas [45], breast, ovarian, prostate or lung cancers [46], [47] but never studied in meningiomas.

In the present study, we investigated the EGFR expression pattern of meningiomas. EGFR isoforms were assessed on IHC with EGFR ECD-Antibody (ECD Ab) that recognized all isoforms and with ICD-Antibody (ICD-Ab) that recognized only isoform a (EGFR full length receptor) and EGFRvIII. We then investigated their corresponding mRNA levels by Quantitative RT-PCR (EGFR v1, v2, v3, v4 and vIII mutant mRNAs). Finally, *EGFR* gene amplification was assessed by Multiplex Ligation-dependant Probe Amplification (MLPA). These data were analyzed with respect to tumor grade and to patient outcome. To our knowledge, it is the first report on meningiomas that compared the difference in IHC staining between ECD and ICD EGFR antibodies and that studied the expression level of mutant EGFRvIII and sEGFR-encoding transcripts.

## Material and Methods

### Patients and Tissue Samples

We studied 69 meningiomas obtained from adult patients undergoing surgery at Limoges Dupuytren University Hospital between 1995 and 2009. All samples were used in accordance with French bioethics laws regarding patient information and consent. Ethics approval was obtained from the “comité médico-scientifique de la tumorothèque de l’Hôpital Dupuytren”, the bioethics committee of our hospital. Before storage in the tumor bank, samples are anonymized when received in the Pathology Department and only the number of anonymity, age and sex are provided to users. Out of 69 patients, 38 underwent surgery for intracranial meningiomas from 1996 to 2004. In agreement with our bioethics committee’s procedure, no information was given to and no written or verbal consent was obtained from these patients because samples were already collected and referred to research prior the French bioethical law (2004). From 2005, the new French law on Bioethics applies. The law states that patients 1) must be informed of the possible use of their samples for research purposes 2) should not have expressed their refusal to the use of their samples for research purposes. However, the law does not specify whether the information or the refusal must be collected verbally or in a written form. Thus in our Institution, patients who underwent surgery are verbally informed and only in cases of refusal their written opposition is recorded. In that case, samples are no longer eligible for research purposes. This procedure has been validated and implemented by the bioethics committee of our Institution.

Clinical, radiological, therapeutic and survival data were obtained by a retrospective query. In 67 cases, surgical resection could be evaluated according to the Simpson grade which was determined from the surgical records [48]. No patients had received EGFR-targeted chemotherapy. At the time of resection, tumor samples were fixed in 4% formalin, embedded in paraffin and sections were stained with hemalum phloxin saffron. A part of the surgical specimen was snap frozen and stored at −80°C. The histological tumor types and grade of meningiomas were determined according to the WHO classification. Histologically benign meningiomas but presenting a brain invasion were classified as grade II [2]. In cases where a preoperative embolization was performed (height tumors), we interpreted the presence of necrosis as secondary to embolization and not as true tumor necrosis.

**Figure 1:**
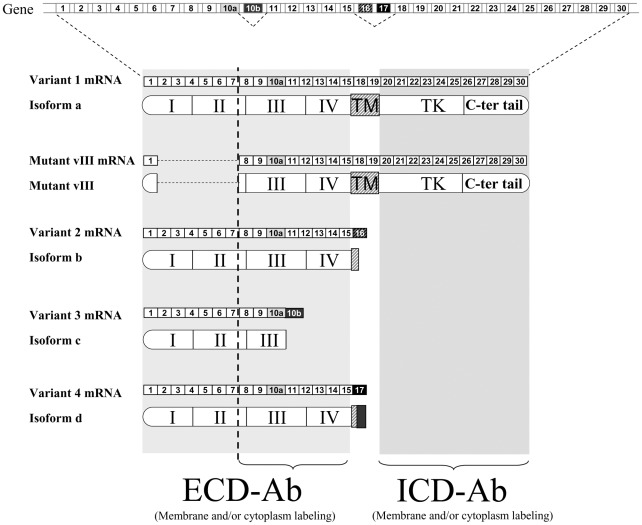
ECD-Ab and ICD-Ab targeting. *EGRF* gene contains 30 exons and generates 5 different mRNAs. Variant 1 mRNA encodes the whole EGFR isoform a. Alternative splicing generates variant mRNAs 2, 3, and 4 that encode sEGFR isoforms b, c and d respectively. EGFRvIII mutant mRNA with a 801 bp (exons 2–7) deletion produces EGFR vIII that lack amino acids 2 to 273. ECD-Ab targets EGFR extracellular domain and recognizes sEGFR and vIII mutant, whereas ICD-Ab targets EGFR intracellular domain and recognizes EGFR isorform a and vIII mutant.

### Quantitative RT-PCR

#### Total RNA extraction

Before RNA extraction, hemalum phloxin-stained sections of the frozen tissue were prepared and examined to ensure that the tissue samples were located in representative areas of the tumors. Tumor tissue (5 to 30 mg) was incubated with 1 mL Qiazol® solution (Qiagen, Courtaboeuf, France) and CK14 ceramic beads (Ozyme) and pulverized two times for 40 sec each at 6,500 rpm in Precellys 24 homogenizer (Ozyme). Homogenized tissues were lysed and RNA purification was performed according to the manufacturer’s protocol (“RNeasy lipid tissue kit”, Qiagen). A DNase I digestion step was included for each extraction to prevent contamination of the RNA by genomic DNA. RNA concentration and purity was estimated by spectrophotometry (NanoDrop ND1000, Labtech France). RNA quality was assessed by capillary electrophoresis (Bioanalyzer 2100, Agilent Technologies) and only RNAs with an integrity number >6 were used for analysis. Eight patients were excluded from analysis due to the absence of tumor tissue or to RNA degradation after extraction.

### Quantitative and Qualitative RT-PCR

Complementary DNA (cDNA) was synthesized from 2 µg of total RNA using the Transcriptor First Strand cDNA Synthesis® kit (Roche) and hexamer primers according to manufacturer’s protocol. Primers sequence and locations were carefully selected to amplify EGFRv1-vIII, EGFRv2, EGFRv3, EGFRv4, EGFRvIII and hypoxanthine phosphoribosyl transferase (HPRT) as previously described [5]. For quantitative PCR of v1, v2, v3, v4 transcripts, HPRT was used to normalize results, based on previous comparative experiments. Specific amplification of EGFRvIII mRNA was considered as qualitative for the following reason: the 5′ forward primer was designed to overlap exon1-exon8 boundary for specificity purpose but PCR generated primer dimers that did not allow accurate quantification. However, on the PCR fusion profile, EGFRvIII specific products were detectable down to 10 copies. PCR were performed on a Rotor Gene thermocycler (Corbett Research) using the “Light Cycler Fast Start DNA Master SYBR Green I” kit (Roche). All targets were amplified in the presence of 3 mM MgCl_2_ and 0.5 µM primers. The mRNA levels were quantified using the ΔΔCt method ((Ct_sample_−Ct_calibrator_)_interest gene_−(Ct_sample_−Ct_calibrator_)_reference gene_), modified according to Pfaffl [49], with efficiency correction by the Rotor Gene Software, and were expressed in relative arbitrary units (R.A.U.). For each interest gene, values were normalized as follows: Sample_x_ mRNA rate  =  Raw Sample_x_ mRNA rate/mean_1→n_ raw mRNA rate.

**Figure 2:**
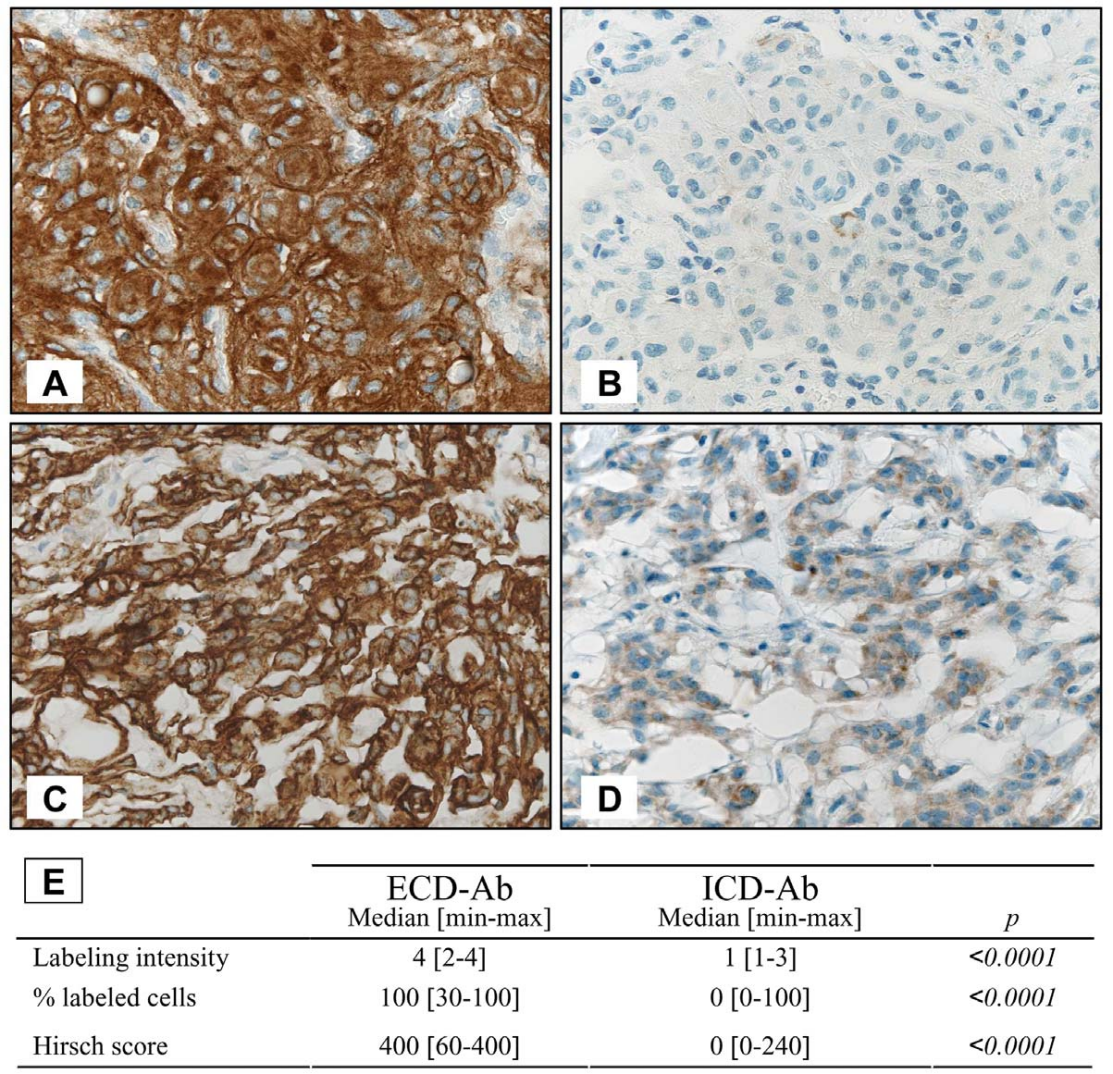
EGFR immunohistochemistry. (A and B) Grade I transitional meningioma and (C and D) grade II chordoid meningioma: tumor cells showed a very strong labeling, membranous and cytoplasmic, with ECD-Ab (A and C) while rare cells were labeled, mainly within the cytoplasm, with the ICD-Ab (B and D); (E) Labeling parameters are presented for ECD-Ab and ICD-Ab.

### Multiplex Ligation-Dependent Probe Amplification (MLPA) Procedure and Data Processing

EGFR gene amplification was studied using the MLPA technique with the SALSA P105 (MRCHolland, http://www.eurogentest.org/uploads/1247475007479/MLPA_validation report_TJ_version1_2009 0710.pdf). MLPA was performed on 35/69 patients randomly selected in grade I (14/29) and grade II (14/33) tumors, and in all grade III meningiomas (7/7). Four normal control DNA samples, isolated from blood of healthy volunteers, were included in MLPA experiment and for data processing. Probes contained in MLPA SALSA P105 hybridized 11 of the 30 exons of EGFR gene, and also contained 8 control probes located on chromosome without known abnormalities in meningiomas. Probe sequences and location are available on the manufacturer site.

MLPA was performed as described by the manufacturer with minor modifications. Briefly, DNA (30 ng) was dissolved in 5 µl of TE-buffer (10 mmol/L Tris, pH 8.2, 1 mmol/L ethylenediaminetetraacetic acid, pH 8.0), denatured, and subsequently cooled to 25°C. After adding the probe mix, the sample was denatured, and the probes were allowed to hybridize (16 hours at 60°C). After ligation of both probe pairs and inactivation of ligase, PCR was performed in a volume of 50 µl containing 10 µl of the ligation reaction mixture using the PTC 200 thermal cycler (MJ Research Inc., Waltham, MA) (35 cycles of denaturation at 95°C for 30 seconds, annealing at 60°C for 30 seconds, and extension at 72°C for 1 minute with a final extension of 20 minutes at 72°C). Fragments were separated and quantified by electrophoresis on an ABI 3130 XL capillary sequencer (Applied Biosystems, Foster City, CA) and Genemapper analysis (Applied Biosystems).

Data analysis was performed in Excel using a “in house” method modified from Jeuken et al., 2006 [50] : first, the fraction of each peak was calculated by dividing the peak area value of each probe amplification product by the combined value of the control probes within the sample, to compensate for PCR efficiency of the individual samples. Subsequently this relative peak value or so-called probe fraction is divided by the mean probe fraction of this fragment within the included reference DNAs, generating the normalized peak value or the so-called probe ratio.

Values obtained for six negative samples (blood samples N°1–3 and glioma samples N°4–6 with unamplified EGFR) were used as reference to set theoretical threshold [51] at 0.5 to identify DNA losses and at 2 for DNA gains. Based on values obtained for a glioma sample with an amplified EGFR (sample N°8), polysomy or amplification was concluded when values above 2 was reached for all EGFR exons.

**Figure 3:**
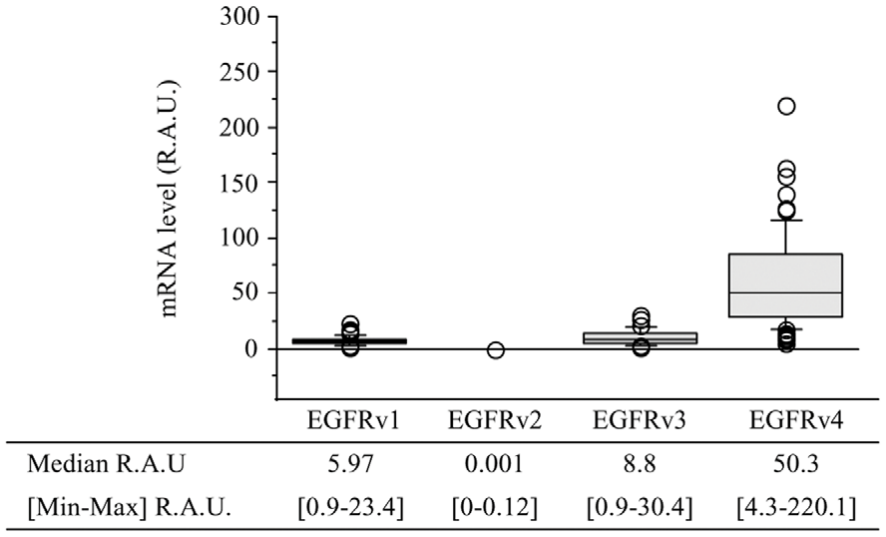
Expression of EGFR variant mRNA. EGFR v1, v2, v3 and v4 mRNA levels were expressed in R.A.U.

### Immunohistochemistry

After representative areas of tumor tissue were selected using hemalum phloxin saffron-stained sections, five µm-thick sections were cut from paraffin-embedded blocks. Sections were incubated with Ki67 antibody (clone MiB-1, DakoCytomation, Glostrup, Denmark; 1/200^e^), EGFR ECD-Ab (clone 3C6, Ventana Medical System, France; pure) and EGFR ICD-Ab (clone EGFR.25, Novocastra Laboratories, United Kingdom; 1/500^e^), (Figure 1). Sample slides were processed automatically (BenchMark XT ICH/ISH, Ventana Medical Systems, USA) according to protocols supplied by the antibody manufacturers. For each section, we recorded the type of cell expressing EGFR protein, the percentage of labeled cells, the labeling intensity and the resulting Hirsch score as previously described [52], [53], [54], [55], [56], [57]. For Ki67 labeling index, 10% of labeled cells was taken as a cut-off value for analyses.

### Statistical Analyses

Statistical analyses were performed with StatView® 5.0 (SAS Institute, Inc., Cary, NC, U.S.A.) and PAST 2.08 b (http://folk.uio.no/ohammer/past, [58]) software. A correlation between quantitative variable was assessed with Pearson ρ test. Fisher’s exact test was used to assess differences between nominal variables. Means variations according to variables were compared with the Student-t test. Progression free survival (PFS) and overall survival (OS) were analyzed by Kaplan-Meier and median PFS or OS medians were compared with the non-parametric logrank. Results for which p<0.05 were considered to be statistically significant.

**Figure 4:**
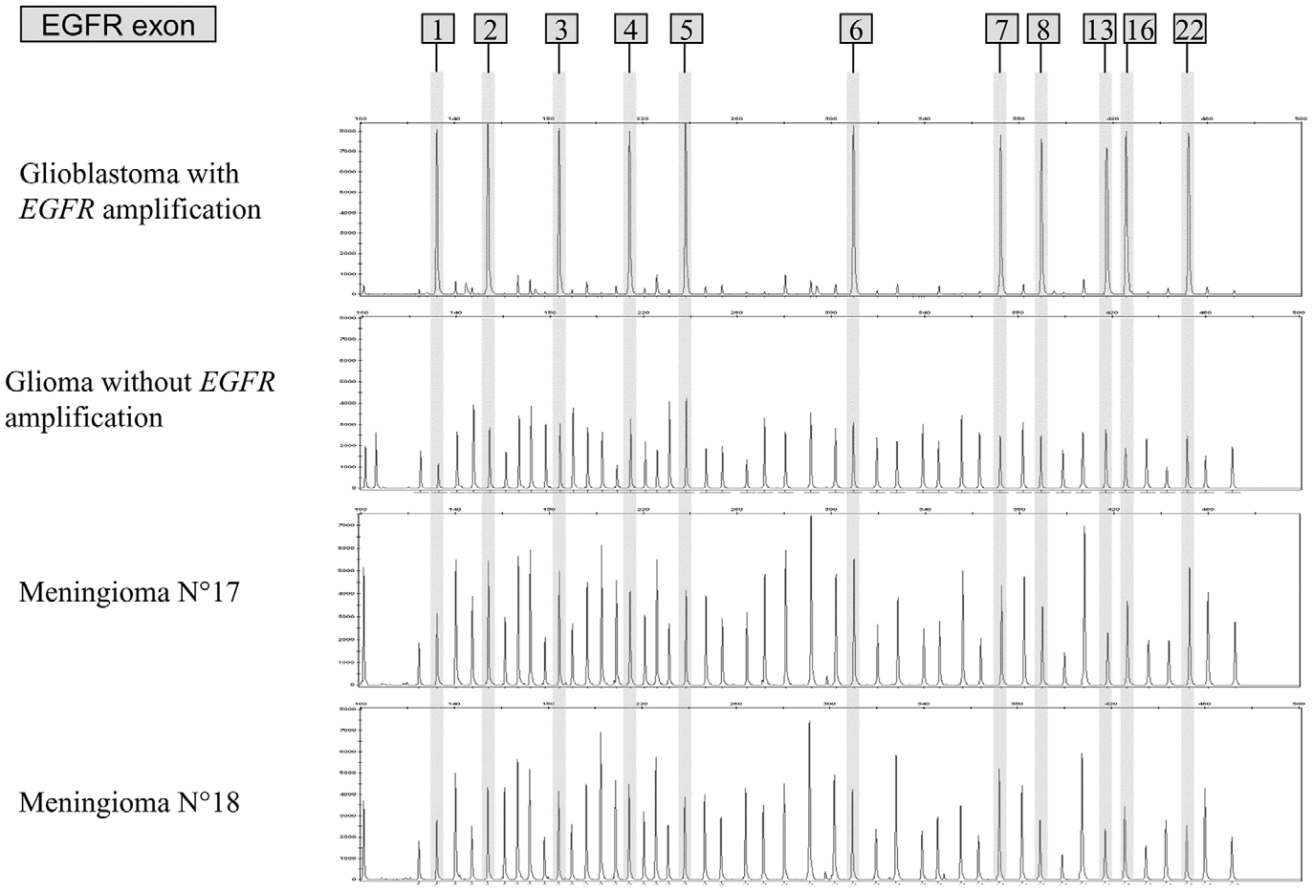
EGFR gene amplification. Genomic DNAs from glioma with and without EGFR amplification were used as positive and negative controls respectively. Two examples of results obtained for meningioma are presented.

## Results

### Tumor Characteristics

Histopathological features of the 69 meningiomas are given in Table 1. Tumors locations were cerebral convexity (n = 39), falx (n = 5), parasagittal (n = 5), paratentorial (n = 1) regions and skull base (n = 19). They were not related to tumor grade (data not shown). Relationship between demographical data, histological grade, Simpson grade, presence or absence of tumor necrosis and Ki67 labeling index is indicated in Table 2.

**Table 1:** Distribution according to histopathological type and grade.

	All	Grade I^a^		Grade II^a^		Grade III^a^	
		w. brain inv.	w/o. brain inv.	w. brain inv.	w/o. brain inv.	w. brain inv.	w/o. brain inv.
**n**	69	0	29	14	19	5	2
**Tumor histology**							
Microcystic	1	–	1	–	–	–	–
Fibrous	9	–	9	–	–	–	–
Transitional	18	–	13	5	–	–	–
Meningothelial	14	–	6	8	–	–	–
Clear cells	1	–	–	–	1	–	–
Atypical	12	–	–	1	11	–	–
Chordoid	7	–	–	–	7	–	–
Malignant	7	–	–	–	–	5	2

a Tumors with or without brain invasion (w. brain inv. and w/o. brain inv.) were distinguished.

**Table 2:** Relationship between tumor grade, demographic data, Simpson grade, absence or presence of tumor necrosis and Ki67 labeling index.

	All	Grade I	Grade II	Grade III	*p*
**N**	69	29	33	7	
**Age** (median)	56.8	57	58.2	48.2	*<0.05*
**Sex**					
Men	26	7	13	6	*0.01*
Women	43	22	1	1	
**Simpson Grade**					
1	22	8	13	1	*0.1*
2	36	19	14	3	
3	4	1	3	0	
4	5	1	2	2	
n.a.	2	-	1	1	
**Tumor Necrosis**					
Yes	18	3	9	6	*0.0008*
No	43	22	20	1	
Preoperative embolization	8	4	4	-	
**Ki67 Labeling Index**					
<10%	55	29	25	1	*<0.0001*
≥10%	14	0	8	6	

### Immunohistochemical Detection of EGFR Proteins

ECD-Ab and ICD-Ab targeted extracellular and intracellular EGFR domain respectively (Figure 1). Labeling localizations were slightly different according to manufacturer indications (Figure 1). ECD-Ab stained membrane and cytoplasm of tumor cells (Figure 2 A and C) while staining with ICD-Ab was almost always restricted to the cytoplasm (Figure 2 B and D). In three cases, a staining was observed in both endothelial and tumor cells with the ECD-Ab.

**Figure 5:**
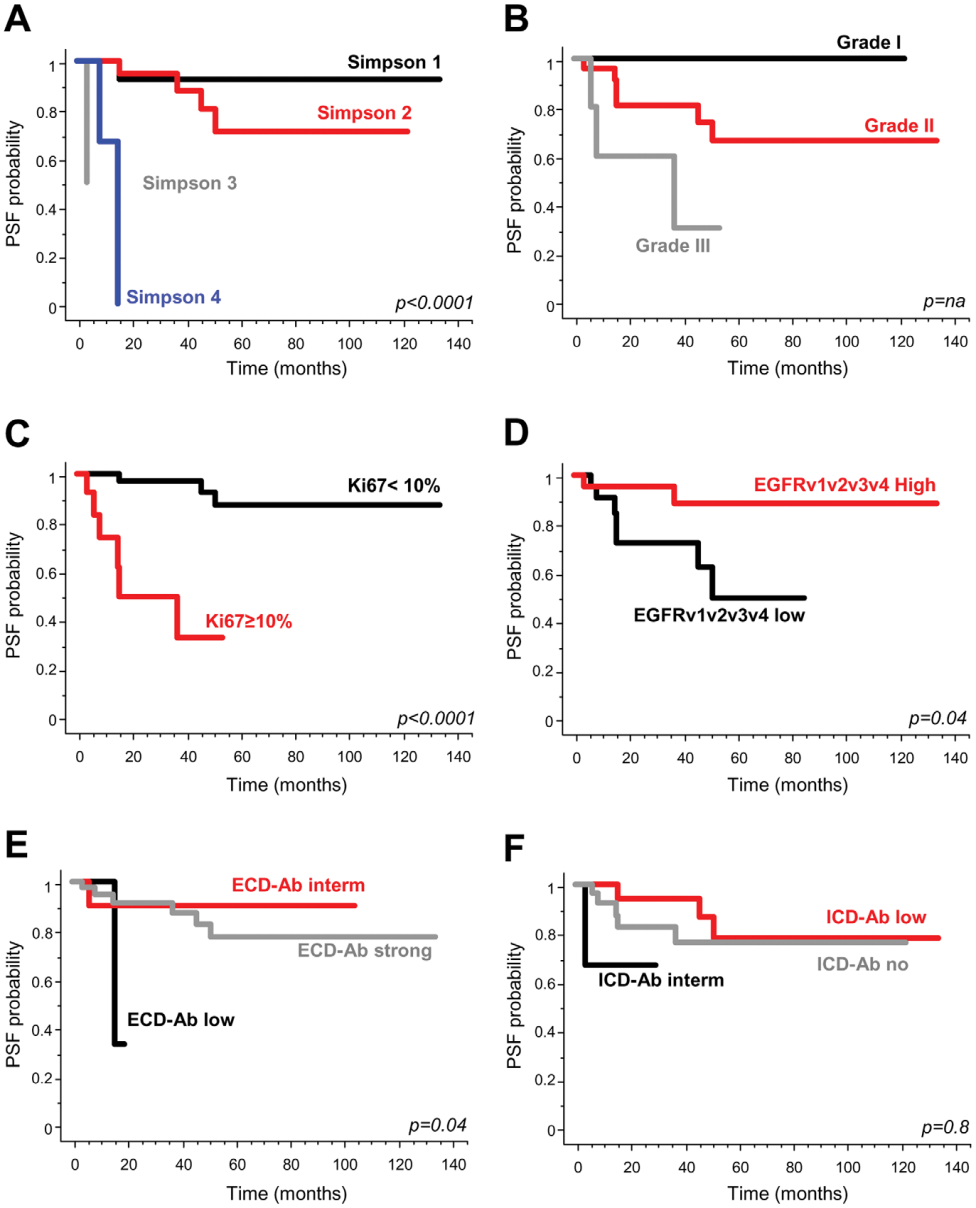
Patient PFS according to clinical and biological parameters. Probability of progression free durvival (PFS) was expressed in months, according to Simpson grade (A), histological tumor grade (B), Ki67 labeling index (C), EGFRv1v2v3v4 mRNA levels (D), ECD-Ab labeling (E), and ICD-Ab labeling (F).

As shown on immunohistochemical sections (Figure 2A, B, C, D), staining intensity, percentage of stained cells and resulting Hirsch Scores were significantly higher for ECD-Ab than those obtained with ICD-Ab (p<0.0001, Figure 2E). All meningiomas were labeled with ECD-Ab, 78% (54/69 cases) of them having a strong labeling (Table 3). In contrast, only 45% of the tumors were labeled with ICD-Ab and all of them presented only low or intermediate labeling. All but one tumors stained with ICD-Ab had intermediate or strong ECD-Ab staining, whereas tumors with low or intermediate ECD-Ab labeling were not or very lightly stained with ICD-Ab.

**Table 3:** Comparison for ECD-Ab and ICD-Ab score labeling.

		ECD-Aba			
		+++	++	+	No
**ICD-Aba**	+++	–	–	–	–
	++	3	–	–	–
	+	24	3	1	–
	No	27	8	3	–

a ECD-Ab and ICD-Ab labeling was expressed as Hirsch score: strong (+++, score 301–400), intermediate (++, score 201–300), low (+, score 1–200), no expression (No, score 0).

ECD-Ab and ICD-Ab labeling score was not associated with patient age, sex, absence or presence of tumor necrosis, brain invasion and tumor grade (Table 4). We observed that ICD-Ab labeling score tended to be lower in Simpson grade 1 and 2. ECD-Ab labeling was inversely related to Ki67 labeling index (p = 0.01) (Table 4).

**Table 4:** Relationship between immunohistochemical EGFR detection and clinicopathological parameters.

	n	ECD-Ab Hirsch Score		ICD-Ab Hirsch Score	
		Mean ± SD	*p*	Mean ± SD	*p*
**All**	69	367.7±73.4		23.5±58.8	
**Sex**					
Male	26	366.5±79.6	*>0.99*	28±70.7	*0.4*
Female	43	366.7±69.9		16±43.8	
**Age (years)**					
≤56.8	34	360.9±73.6	*0.5*	18.5±50.6	*0.8*
>56.8	35	372.6±73.3		22.6±60.4	
**Simpson Grade**					
1	22	354.1±85.3	*0.3 (1 vs.3 or 4)*	17.5±46.2	*0.06 (1 vs. 4)*
2	36	373.1±66.8	*0.4 (2 vs. 1 or 3)*	11.7±39	*0.01 (2 vs. 4)*
3	4	400±0		60±120	*0.08 (2 vs. 3)*
4	5	356±98.4		74±102.9	
**Brain Invasion**					
Yes	19	371.6±71.3	*0.7*	1.8±4.5	*0.08*
No	50	364.8±74.4		27.6±63.6	
**Tumor Necrosis**					
Yes	43	356.7±90.3	*0.6*	31±69.2	*0.4*
No	18	369.3±70.3		18.4±53.5	
**Histological Grade**					
I	29	382.8±38.4	*0.1 (I vs. II)*	23.2±52.9	*0.3 (I vs. III)*
II	33	355.2±91.1	*0.2 (I vs. III)*	22.4±62.9	*0.4 (II vs. III)*
III	7	354.3±85.4		0.6±1	
**Ki67 Labeling Index**					
<10%	55	377.8±56.2	*0.01*	17.3±46.9	*0.3*
≥10%	14	322.9±110.6		33.3±81.5	

### EGFRv1, v2, v3, v4 and EGFRvIII Mutant mRNA Levels and their Association with other Tumor Variables

All meningiomas expressed EGFR v1, v3 and v4 mRNA whereas only 50% of the tumors expressed EGFRv2 mRNA. Results indicated the following rank of magnitude EGFR v4> EGFR v3> EGFRv1> EGFR v2 (Figure 3). Moreover, except for EGFRv2 mRNA, each EGFR variant mRNA levels were straightly correlated with each other (p<0.0001, data not shown). The EGFRvIII mutant mRNA was never detected.

There was no significant association between any variant mRNA levels with demographic variable, Simpson grade, brain invasion, tumor type and grade or Ki67 labeling index (results not shown).

### MLPA Results for EGFR Amplification

MLPA results are given in the Table 5. Examples of the MLPA profiles are presented in Figure 4. Values <0.5 or >2 did not reflect a loss or a gain when found only on certain exon and were considered as not interpretable, probably due to variability in DNA quality. Compared to a glioma sample with an amplified *EGFR*, none of the analyzed meningiomas showed *EGFR* gene amplification.

**Table 5:** MLPA results for EGFR gene amplification.

N°	Type	Ex 1	Ex 2	Ex 3	Ex 4	Ex 5	Ex 6	Ex 7	Ex 8	Ex 13	Ex 16	Ex 22
1	Negative control	1.13	0.90	0.91	1.36	1.03	0.51	0.82	0.84	1.01	1.40	1.37
2	Negative control	1.55	0.83	1.00	1.31	1.13	0.50	0.91	0.98	0.94	1.79	1.34
3	Negative control	1.46	1.08	0.98	1.66	1.03	0.38	0.94	1.01	0.88	1.59	1.41
4	Negative control	1.62	1.92	1.54	1.88	1.39	0.60	1.48	1.34	1.70	1.75	2.19
5	Negative control	1.21	0.67	0.74	1.32	1.04	0.84	0.82	1.23	1.00	1.28	1.05
6	Negative control	1.27	2.34	1.57	1.89	1.24	0.66	1.63	1.74	1.78	1.65	1.74
7	Positive control	**32.47**	**30.97**	**32.07**	**33.89**	**28.50**	**26.44**	**26.05**	**35.51**	**28.88**	**31.53**	**30.17**
8	Grade I	1.83	1.33	1.24	1.41	1.28	0.61	1.07	1.23	1.22	1.87	1.50
9	Grade I	1.47	0.55	0.80	1.21	0.92	0.50	0.93	1.07	0.95	1.17	0.89
10	Grade I	1.59	0.56	0.75	1.11	1.10	0.77	1.04	1.24	1.27	1.30	0.74
11	Grade I	1.35	0.67	0.74	1.09	1.00	0.57	0.83	1.04	1.00	1.20	0.90
12	Grade I	1.34	0.75	0.70	1.09	1.10	0.64	0.90	0.99	0.95	0.99	0.87
13	Grade I	1.42	0.60	0.75	1.07	1.17	0.92	0.98	0.92	0.99	1.24	1.06
14	Grade I	1.49	0.62	0.61	0.89	0.88	0.48	0.73	0.71	0.83	1.00	1.15
15	Grade I	0.75	0.35	0.61	0.57	0.73	0.40	0.48	0.64	0.58	0.63	0.58
16	Grade I	1.75	0.65	0.91	1.22	1.28	0.72	0.98	1.04	0.93	1.31	1.36
17*	Grade I	1.08	0.61	0.87	1.31	1.02	0.64	0.93	1.00	0.89	1.39	0.84
18*	Grade I	1.26	0.62	0.68	0.96	0.84	0.54	0.78	0.91	0.93	0.65	0.75
19	Grade I	1.53	0.79	1.10	1.62	1.24	0.75	1.05	1.31	1.19	1.31	1.31
20	Grade I	1.12	1.07	0.84	1.24	1.05	0.60	0.79	0.98	0.83	1.42	1.33
21	Grade II	1.36	0.61	0.72	1.21	1.00	0.71	0.96	0.89	0.99	1.15	0.96
22	Grade II	1.58	0.48	0.67	0.83	1.11	0.89	0.95	1.13	1.20	1.01	0.97
23	Grade II	1.56	0.61	0.86	1.31	1.29	0.81	1.23	1.23	1.30	1.34	1.13
24	Grade II	2.45	1.22	1.27	1.89	1.79	1.29	1.62	1.69	1.89	1.46	1.83
25	Grade II	1.35	0.52	0.83	1.19	1.35	0.85	1.04	1.24	1.37	0.93	1.21
26	Grade II	1.40	0.46	0.63	0.84	0.89	0.50	0.81	0.79	1.04	0.92	0.92
27	Grade II	1.04	0.32	0.45	0.72	0.70	0.39	0.63	0.74	0.60	0.67	0.59
28	Grade II	1.46	0.67	0.86	1.30	0.93	0.57	0.93	1.07	0.97	1.15	1.10
29	Grade II	1.67	0.63	1.01	1.25	0.89	0.79	1.04	1.20	0.88	1.33	0.94
30	Grade II	1.25	0.52	0.75	1.07	0.83	0.88	1.00	1.17	1.19	0.93	0.85
31	Grade II	1.47	0.90	0.99	1.35	1.05	0.84	1.06	1.25	1.27	1.43	1.05
32	Grade II	1.52	0.64	1.17	1.35	1.10	0.92	1.15	1.47	1.11	1.17	1.09
33	Grade II	1.27	0.80	1.07	1.29	1.00	0.77	0.89	1.31	1.17	1.22	1.09
34	Grade II	1.37	0.55	0.87	0.99	1.05	1.11	1.17	1.50	1.27	0.93	0.86
35	Grade II	2.23	0.77	0.93	1.24	1.43	1.86	2.18	2.75	2.22	1.57	1.12
36	Grade III	0.76	0.37	0.34	0.73	0.64	0.48	0.63	0.56	0.61	0.67	0.44
37	Grade III	1.02	0.34	0.52	0.81	1.12	0.83	1.03	0.90	1.16	0.93	0.92
38	Grade III	0.76	0.30	0.62	0.88	1.33	1.24	1.14	1.02	0.86	0.96	0.99
39	Grade III	1.35	0.74	0.74	1.34	0.95	0.67	1.09	1.27	1.05	0.85	0.95
40	Grade III	2.18	1.12	1.09	1.94	1.47	1.00	1.58	2.01	1.64	1.87	1.33
41	Grade III	2.47	1.12	1.36	1.77	1.90	1.39	1.95	2.48	1.71	1.51	1.61
42	Grade III	1.10	0.77	0.97	1.20	0.86	0.64	0.88	1.43	0.85	0.75	0.80

Negative controls were healthy volunteers blood (N°1–3) or glioma with no EGFR amplification as validated by a standard FISH technic (N°4–6). Positive control was glioblastoma harboring EGFR amplification as validated by a standard FISH technic (N°7). *Cases N°17 and 18 were chosen to illustrate MLPA results on figure 4. Lack of amplification was concluded for 2>Ratio<0.5, and amplification for ratio >3 (bold values). 2<Ratio>0.5 were considered as not interpretable (underlined values).

### Relationship with Tumor Progression Free Survival and Overall Survival

At the time of our analysis, the tumors of nine patients had recurred (out of 69) and were all grade III meningiomas. PFS was significantly better for women and for patients having Simpson grade 1 and 2 tumors, grade I *vs.* grade II *vs.* grade III tumors and for meningiomas with a KI67 labeling index less than 10% (Table 6; Figure 5A, B, C). Taken individually, the expression of each mRNAs variant was not significantly link to PFS (Table 6). However, when mRNAs variant expression was analyzed as a whole, high levels were correlated with a better PFS (Figure 5D; Table 6). A better PFS were also found with patients whose tumors were strongly labeled with ECD-Ab. No such link could be drawn with ICD-Ab (Figures 5E and 5F respectively). Regarding OS, Simpson grade 1 and 2, absence of brain invasion, grade I and II, and Ki67 labeling index less than 10% were related with a better prognosis (Table 6).

**Table 6:** OS and PFS according to clinical, pathological and molecular parameters.

	**PFS** a			**OS** b	
	Median (months)		*p*	Median (months)	*p*
**Age**					
≤56.8/>56.8	nr/nr		*0.82*	nr/nr	*0.3*
**Gender**					
Male/Female	45.8/nr	*0.002*		nr/nr	*0.4*
**Simpson Grade**					
1/2/3/4	nr/nr/3.6/15.2	*<0.0001*		nr/nr/nr/19.2	*0.03*
**Brain Invasion**					
No/yes	nr/nr	*0.67*		nr/nr	*0.003*
**Histological Grade**					
I vs. II vs. III	nr/nr/37	*n.a.*		nr/nr/19.2	*0.01*
**Ki67 Labeling Index**					
<10% vs. ≥10%	nr 15.54	*<0.0001*		nr nr	*0.03*
**ECD-Ab (Hirsch Score)**					
Low vs. Interm vs. High	15.9/nr/nr	*0.04*		19.2/nr/nr	*n.a.*
**ICD-Ab (Hirsch Score)**					
No vs. Low vs. Interm	nr/nr/nr	*0.2*		nr/nr/nr	*n.a.*
**EGFR variant mRNA Levels** c					
EGFRv1 Weak vs. Strong	nr/nr	*0.5*		nr/nr	*0.4*
EGFRv2 Weak vs. Strong	nr/nr	*0.9*		nr/nr	*0.5*
EGFRv3 Weak vs. Strong	nr/nr	*0.1*		nr/nr	*0.5*
EGFRv4 Weak vs. Strong	nr/nr	*0.4*		nr/nr	*0.1*
ΣEGFRv1v2v3v4 Weak vs. Strong	51.3/nr	*0.04*		nr/nr	*0.5*

a Progression free survival (PFS).

b Overall survival (OS).

c Median values were used as cut-off to determinate weak and strong levels for each variant or sum (Σ) of all variants.

## Discussion

Epidermal Growth Factor Receptor is involved in many tumors, with alterations ranging from protein overexpression, gene amplification, and gene mutation. *EGFR* is also alternatively spliced giving rise to four different isoforms, three of which lacking tyrosine kinase domain. In meningiomas, the second most common intracranial tumor, EGFR expression analysis gave conflicting results particularly on IHC, depending on the antibody used. Hence, the usefulness of EGFR as a potential prognosis or theragnosis marker is questionable.

In this study we described the EGFR-based profiling of meningiomas. EGFR was analyzed at protein, genetic, and transcriptomic levels. Results were confronted to histological and clinical data.

In a 69 meningiomas series, we showed that IHC analysis revealed an ECD-Ab staining significantly stronger than that with the ICD-Ab. Whereas ICD-antibodies only target whole EGFR and the EGFRvIII mutant, the ECD-antibodies also detect sEGFR like isoforms b, c and d as shown by Halle and coworkers in cervical cancers [28]. Our IHC results suggest that meningiomas express other EGFR isoforms than the whole receptor, but do not indicate which ones. However, transcriptional analysis of EGFR variants indicated that all isoforms were present in our series (see below).

Our findings clearly show that IHC data should be interpreted according to the antibody used to investigate EGFR expression. Antibodies directed against the EGFR ECD do not recognize the same isoforms as antibodies directed against the ICD and this might explain the discrepancies found in the literature concerning EGFR expression. EGFR has been reported to be expressed in meningiomas at frequencies greater than 60% [31], [34], [35], [36], [37], [38], [39] and even as high as 100% [59], which is similar to our results with ECD-Ab but not with ICD-Ab. These findings are not only reported in meningiomas but have also been described in other tumor types and with other EGFR antibodies [5], [28], [30], [60], [61], [62].

In addition to whole EGFR, tumor cells express sEGFR proteins that can be generated by alternative mRNA splicing events [13], [14], [15], [16], [18], via proteolytic cleavage of the receptor [11], [12], [13], [20] or by aberrant translocation events [21]. In our series, we found that meningiomas expressed the alternatively spliced mRNA transcripts v2, v3 and v4. That suggests that the EGFR b, c and d isoforms are likely to be produced by meningiomas and supports our immunohistochemical results. These results suggest that the EGFR signaling pathway, including EGFR mRNA variant expression, could be involved in meningiomas oncogenesis. However, we did not find any specific mRNA expression pattern that correlated with the histological tumor type and grade.

*EGFR* gene amplification, associated or not to EGFRvIII mutant expression, is frequently observed in other tumors like gliomas [5], [52], [63], [64], [65], [66], lung cancers [67], [68], breast [69], and prostate adenocarcinomas [70]. *EGFR* gene amplification and EGFRvIII transcripts were not detected in our series, indicating that they are not involved in meningiomas oncogenesis.

Regarding the roles of the EGFR pathway in oncogenesis, the expression pattern of this receptor in meningiomas seems somewhat paradoxical. The EGFR pathway is known to play important roles in cell proliferation, resistance to apoptosis, adhesion, motility, invasion and angiogenesis, all of which are characteristic features of tumor progression. Consequently, EGFR overexpression in many tumors, such as head and neck carcinomas, is associated with malignancy and more aggressive phenotypes [71]. These characteristics represent the basis of anti-EGFR targeted therapies used in clinical oncology. Conversely, in our series of meningiomas, high levels of EGFR expression were associated with a better clinical outcome, as previously reported [31], [32]. Physiopathological mechanisms of EGFR expression remain poorly understood and different hypotheses have been proposed. It has been suggested that the EGFR pathway in meningiomas is stimulated by an autocrine/paracrine mechanism that occurs in association with other control systems [31], [72]. The ultimate result is that EGFR pathway activity is either positively or negatively regulated. Thus, activation of the EGFR pathway could represent a first step in meningiomas oncogenesis, whereas transformation in more aggressive tumors and/or the development of primary grade III meningiomas could result from additional oncogenic mechanisms.

The roles of the different EGFR isoforms are largely unknown in tumor pathology. Albitar et al. reported isoform expression in cell cultures of endometrial adenocarcinoma [73]. Some studies indicate that soluble isoforms could regulate EGFR signaling in normal [17] and in tumor tissues [74]. Truncated EGFR isoforms have been shown to decrease cellular proliferation *in vitro* [16], [75]. The mechanisms responsible for growth inhibition could be competitive binding of EGFR ligands by the soluble isoforms [76], [77] and formation of inactive heterodimers between different isoforms, which competitively prevent the formation of functional holoreceptors and reduce intracellular kinase activity [74]. In our series, the presence of EGFR mRNAs suggests that a particular regulatory mechanism of EGFR signaling could exist in meningiomas. Patients whose tumors produced high levels of v1–v4 mRNAs presented a better PFS. Moreover, improved PFS was associated with a strong or intermediate ECD-Ab staining but not with ICD-Ab labeling. That might indicate that truncated isoforms could act as negative regulators.

Prognosis and theragnosis predictive values have been shown for sEGFR levels in blood and in tumor tissues of certain cancers [22], [23], [24], [25], [26], [27], [28], [29]. These isoforms may compete with active, membrane-bound receptors for the binding of therapeutic antibodies, which could thus explain why some EGFR-targeted therapies failed [61], [78], [79]. In meningiomas, intracellular EGFR inhibitors, like Gefitinib or Erlotinib have been shown to be inefficient [43]. Regarding our results, this could be due to an overexpression of sEGFR rather than entire EGFR isoforms, since these inhibitors are ineffective on EGFR isoforms that lack the ICD.

Finally, several studies have shown that treatment outcomes, with regards to EGFR, are also dependent on serum ligand levels. Several reports showed relation between circulating EGF and patient treatment outcomes in different types of cancers. However, we think that quantifying serum ligand levels goes beyond the scope of our study. First, our study was retrospective and patient serums were not available. We focused our work on the analysis of EGFR isoform expression. Second, the purpose of the present article was to show that like a number of other tumors (lung, ovarian, breast, gliomas), meningiomas expressed EGFR isoforms other than the whole one (isoform a), and this may have important implications in the assessment of EGFR expression, particularly by immunohistochemistry, or for the development of new therapies.

In conclusion, we observed that, in addition to the entire EGFR isoform a (HER1), meningiomas expressed EGFR receptors lacking the ICD that was sustained by the strong expression of the *EGFR* transcripts encoding sEGFR isoforms. No *EGFR* gene amplification was detected and we did not found any mutant vIII expression. Furthermore, a better prognosis was associated to a strong staining with an antibody targeted against the ECD and high *EGFR* transcript levels. This suggests that the oncogenetic mechanisms involving the *EGFR* gene pathway in meningiomas could be different from other tumor types.

### Dupuytren University Hospital Tumor Bank Ethics Committee

Dominique Bordessoule, Hélène Chable, François Denis, Jean Feuillard, Alain Gainant, Isabelle Hérafa, François Labrousse, Boris Melloni, Dominique Mouliès, François Paraf, Pierre-Marie Preux, Nicole Tubiana-Mathieu.
